# Supplementary material for: Differentiation of SARS-CoV-2 naturally infected and vaccinated individuals in an inner-city emergency department
Source: medRxiv. 2021 Oct 14:2021.10.13.21264968. Preprint. [Version 1] doi: 10.1101/2021.10.13.21264968 (PMC8528087; doi:10.1101/2021.10.13.21264968)

medRxiv preprint doi: https://doi.org/10.1101/2021.10.13.21264968; this version posted October 14, 2021. The copyright holder for this preprint (which was not certified by peer review) is the author/funder, who has granted medRxiv a license to display the preprint in perpetuity. This article is a US Government work. It is not subject to copyright under 17 USC 105 and is also made available for use under a CC0 license.

Supplemental Table 1: Characteristics of Utilized Commercial SARS-CoV-2 Assays

Manufacturer	Assay Name	Target antigen (recombinant)	Platform	Manufacturer's Interpretation
Euroimmun, Lubeck, Germany	Anti-SARS-CoV-2 ELISA (IgG)	Spike-1 protein	Manual ELISA	Negative: S/C ratio <0.8<br>Borderline: S/C ratio ≥0.8 & <1.1<br>Positive: S/C ratio ≥1.1
Hangzhou Biotest Biotech Co. Ltd., Hangzhou, China	CoronaCHEK™ COVID-19 IgG/IgM Rapid Test Cassette	Spike RBD	Lateral Flow Assay	IgG and IgM Positive: Three lines appear<br>IgG Positive: Control and IgG lines appear<br>IgM Positive: Control and IgM lines appear<br>Negative: One line in control region<br>Invalid: No control line
Bio-Rad Laboratories, Inc., Hercules, CA, USA	Platelia SARS-CoV-2 Total Ab assay	Nucleocapsid Protein	Manual ELISA	Negative: S/C ratio < 0.8<br>Equivocal: S/C ratio ≥ 0.8 & < 1.0<br>Positive: S/C ratio ≥ 1.0

Abbreviations: ELISA, enzyme-linked immunosorbent assay; S/C, signal to control; RBD, receptor binding domain.

medRxiv preprint doi: https://doi.org/10.1101/2021.10.13.21264968; this version posted October 14, 2021. The copyright holder for this preprint (which was not certified by peer review) is the author/funder, who has granted medRxiv a license to display the preprint in perpetuity. This article is a US Government work. It is not subject to copyright under 17 USC 105 and is also made available for use under a CC0 license.

Supplemental Table 2a: Demographic Characteristics Associated with Natural Infection among 2595 Emergency Department Patients, January –March 2021

Characteristics	Category	Crude OR (95% CI)	Adjusted OR (95% CI)
Age	18-29 years	1.00	1.00
	30-44 years	0.93 (0.70, 1.24)	0.86 (0.64, 1.16)
	45-59 years	**0.77 (0.57, 1.05)**	**0.78 (0.57, 1.07)**
	60-74 years	**0.71 (0.52, 0.98)**	**0.78 (0.56, 1.08)**
	≥ 75 years	0.80 (0.52, 1.24)	0.94 (0.60, 1.46)
Sex	Male	1.00	1.00
	Female	1.06 (0.86, 1.30)	1.04 (0.84, 1.28)
Race	Black	1.00	1.00
	White	**0.73 (0.56, 0.94)**	**0.70 (0.54, 0.91)**
	Other	**2.25 (1.70, 2.96)**	1.24 (0.85, 1.79)
Ethnicity	Non-Hispanic	1.00	1.00
	Hispanic	**4.08 (2.96, 5.63)**	**3.31 (2.16, 5.07)**

Abbreviations: Adj, Adjusted; CI confidence interval; OR, Odds Ratio; n, number; NH non-Hispanic.

medRxiv preprint doi: https://doi.org/10.1101/2021.10.13.21264968; this version posted October 14, 2021. The copyright holder for this preprint (which was not certified by peer review) is the author/funder, who has granted medRxiv a license to display the preprint in perpetuity. This article is a US Government work. It is not subject to copyright under 17 USC 105 and is also made available for use under a CC0 license.

Supplemental Table 2b: Demographic Characteristics Associated with Evidence of Vaccination among 2380 Emergency Department Patients, January –March 2021

Characteristics	Category	Crude OR (95% CI)	Adjusted OR (95% CI)
Age	18-29 years	1.00	1.00
	30-44 years	1.03 (0.70, 1.51)	0.98 (0.67, 1.45)
	45-59 years	**0.48 (0.30, 0.77)**	**0.50 (0.31, 0.80)**
	60-74 years	1.05 (0.70, 1.56)	1.09 (0.73, 1.65)
	≥ 75 years	0.96 (0.55, 1.68)	0.86 (0.49, 1.52)
Sex	Male	1.00	1.00
	Female	**1.36 (1.03, 1.80)**	**1.35 (1.02, 1.80)**
Race	Black	1.00	1.00
	White	**2.30 (1.71, 3.11)**	**2.26 (1.67, 3.07)**
	Other	**2.61 (1.73, 3.91)**	**2.42 (1.51, 3.88)**
Ethnicity	Non-Hispanic	1.00	1.00
	Hispanic	**1.88 (1.13, 3.13)**	1.25 (0.69, 2.29)

Abbreviations: Adj, Adjusted; CI confidence interval; OR, Odds Ratio; n, number; NH non-Hispanic.

medRxiv preprint doi: https://doi.org/10.1101/2021.10.13.21264968; this version posted October 14, 2021. The copyright holder for this preprint (which was not certified by peer review) is the author/funder, who has granted medRxiv a license to display the preprint in perpetuity. This article is a US Government work. It is not subject to copyright under 17 USC 105 and is also made available for use under a CC0 license.

Supplemental Figure 1a. Testing algorithm results on samples from known vaccinated, naturally infected and pre-pandemic samples.

Supplemental Figure 1b. Determination of sensitivity and specificity of vaccinated state in testing algorithm.

Supplemental Figure 1c. Determination of sensitivity and specificity of naturally infected state in testing algorithm.

Supplemental Figure 2a. Testing algorithm results for ED samples collected in 2020.

Supplemental Figure 2b. Testing algorithm results for ED samples collected in 2021.

Supplemental Figure 3a. Euroimmun S/C values of antibody response against spike protein by days post-second dose or confirmed infection. Euroimmun index values were plotted against time since receipt of the second dose of a vaccine (vaccinated samples) or time since confirmed SARS-CoV-2 PCR-positive infection (infected samples and vaccinated/infected samples).

Supplemental Figure 3b. Bio-Rad S/C values of antibody response against nucleocapsid by days post-second dose or confirmed infection. Bio-Rad index values were plotted against time

medRxiv preprint doi: https://doi.org/10.1101/2021.10.13.21264968; this version posted October 14, 2021. The copyright holder for this preprint
(which was not certified by peer review) is the author/funder, who has granted medRxiv a license to display the preprint in perpetuity.
This article is a US Government work. It is not subject to copyright under 17 USC 105 and is also made available
for use under a CC0 license.

since receipt of the second dose of a vaccine (vaccinated samples) or time since confirmed SARS-CoV-2 PCR-positive infection (infected samples and vaccinated/infected samples).

Supplementary Figure 1a.

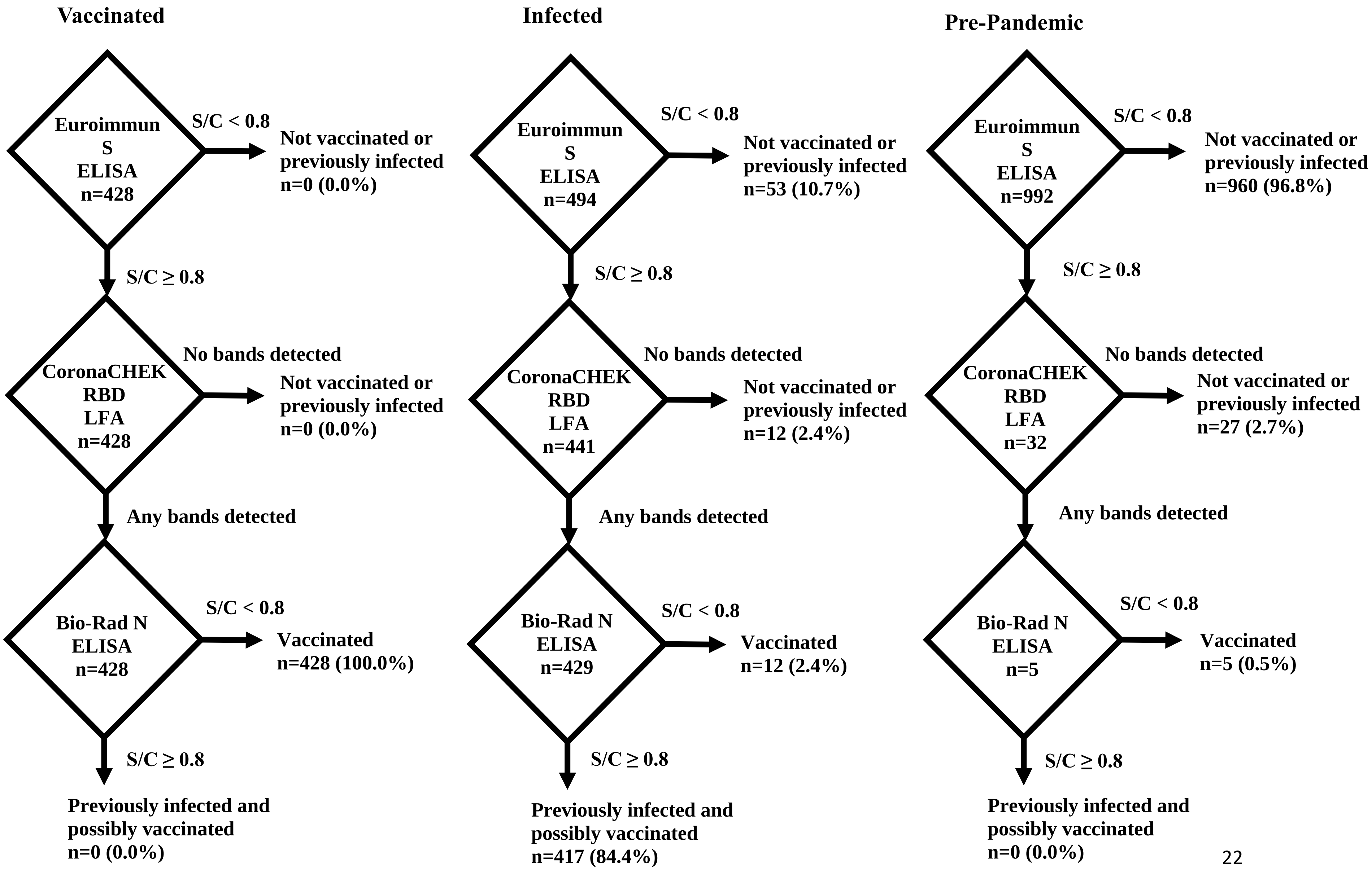

medRxiv preprint doi: https://doi.org/10.1101/2021.10.13.21264968; this version posted October 14, 2021. The copyright holder for this preprint (which was not certified by peer review) is the author/funder, who has granted medRxiv a license to display the preprint in perpetuity. This article is a US Government work. It is not subject to copyright under 17 USC 105 and is also made available for use under a CC0 license.

Supplementary Figure 1b.

	Vaccinated	Not vaccinated
**Euroimmun / CoronaCHEK positive/ BioRad Negative**	**428**	**17**
**Any other outcome**	**0**	**1469**

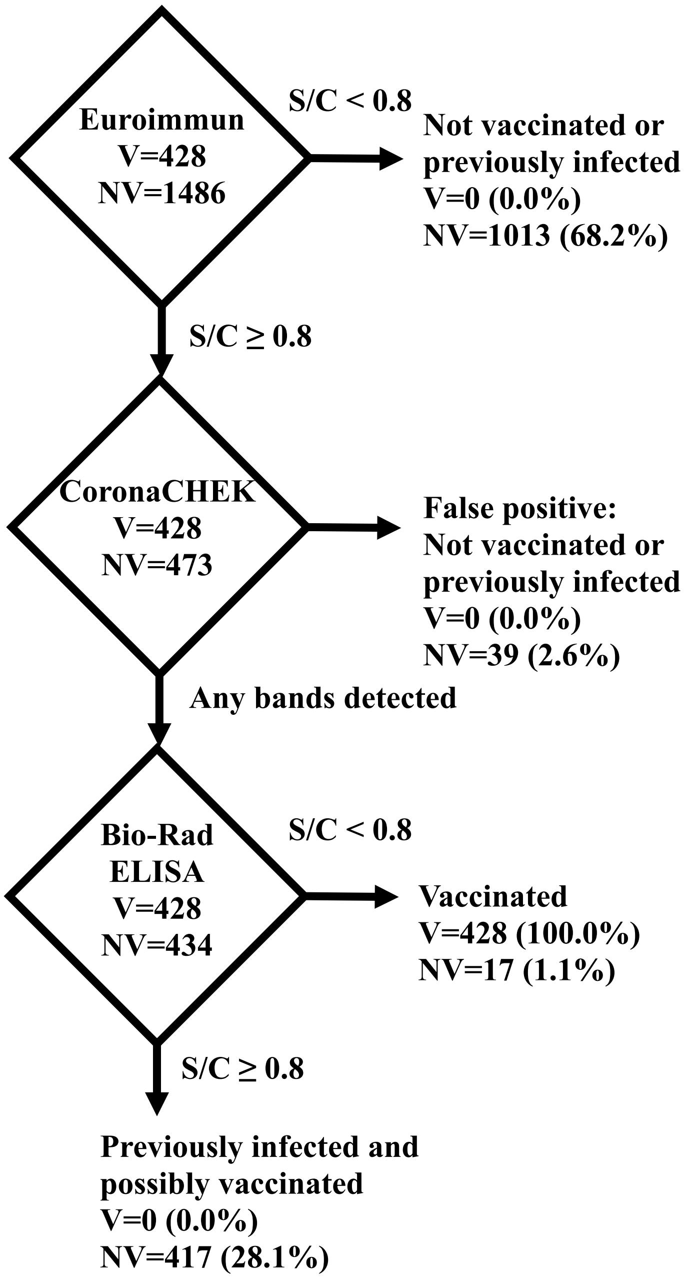

Sample Sets used for the analysis

Vaccinated (V)

Vaccine trial participants (n=68)
Health Care Professionals (n=360)

Not Vaccinated (NV)

Convalescent plasma donators (n=244)
Clinical Characterization Protocol for Severe Infectious Diseases (n=246)
Infected health care professionals (n=4)
Pre-pandemic samples (n=992)

medRxiv preprint doi: https://doi.org/10.1101/2021.10.13.21264968; this version posted October 14, 2021. The copyright holder for this preprint (which was not certified by peer review) is the author/funder, who has granted medRxiv a license to display the preprint in perpetuity. This article is a US Government work. It is not subject to copyright under 17 USC 105 and is also made available for use under a CC0 license.

Supplementary Figure 1c.

	Infected	Not Infected
**Euroimmun / CoronaCHEK positive/ BioRad Positive**	**417**	**0**
**Any other outcome**	**77**	**1420**

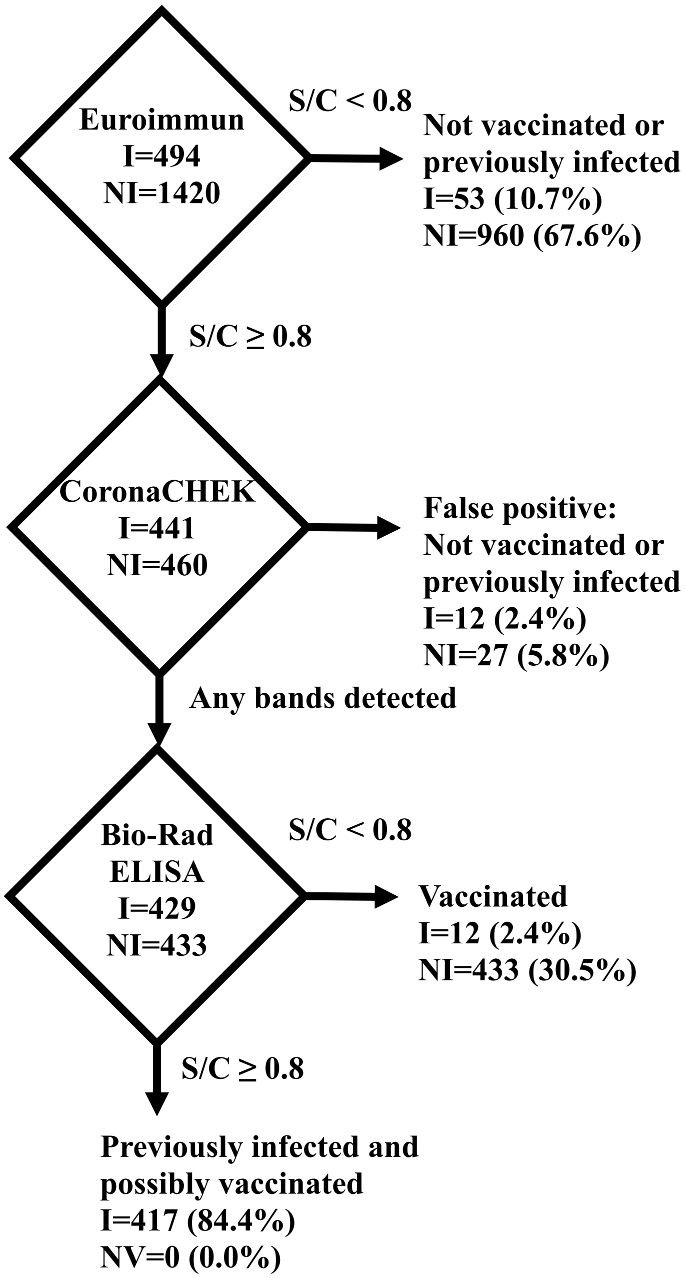

Sample Sets used for the analysis

Infected (I)

Convalescent plasma donators (n=244)
Clinical Characterization Protocol for Severe Infectious Diseases (n=246)
Infected health care professionals (n=4)

Not Infected (NI)

Vaccine trial participants (n=68)
Health Care Professionals (n=360)
Pre-pandemic samples (n=992)

medRxiv preprint doi: https://doi.org/10.1101/2021.10.13.21264968; this version posted October 14, 2021. The copyright holder for this preprint (which was not certified by peer review) is the author/funder, who has granted medRxiv a license to display the preprint in perpetuity. This article is a US Government work. It is not subject to copyright under 17 USC 105 and is also made available for use under a CC0 license.

Supplementary Figure 2a.

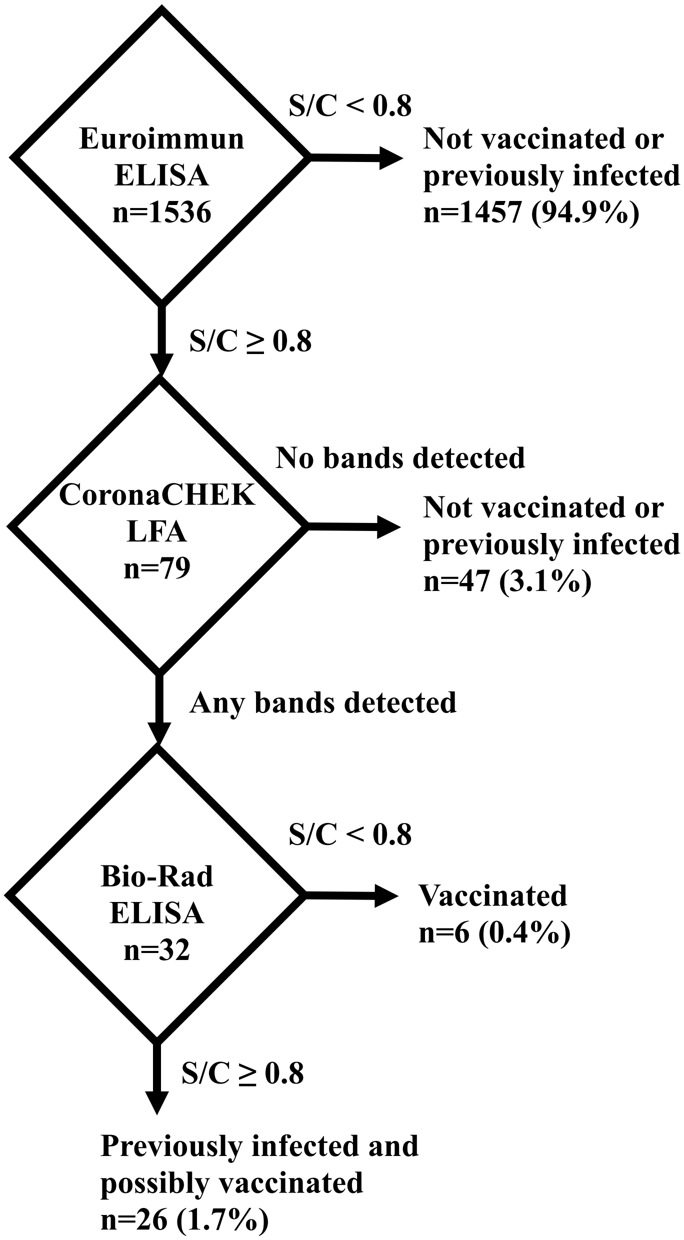

medRxiv preprint doi: https://doi.org/10.1101/2021.10.13.21264968; this version posted October 14, 2021. The copyright holder for this preprint (which was not certified by peer review) is the author/funder, who has granted medRxiv a license to display the preprint in perpetuity. This article is a US Government work. It is not subject to copyright under 17 USC 105 and is also made available for use under a CC0 license.

Supplementary Figure 2b.

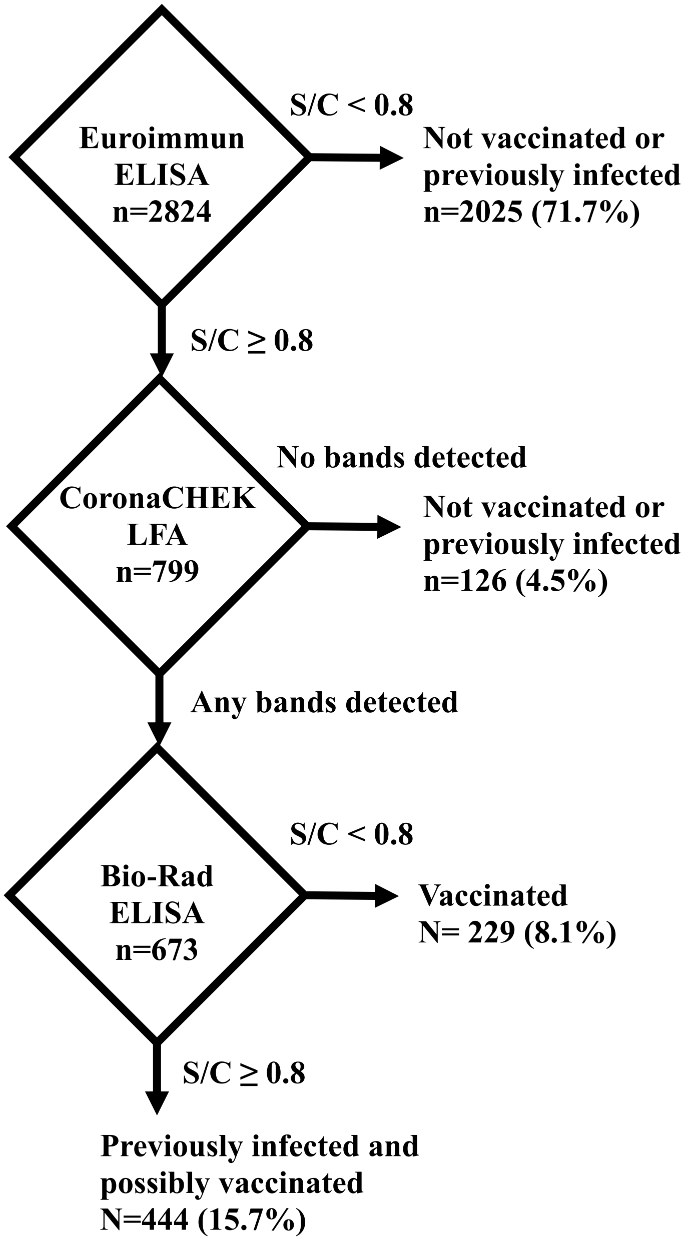

medRxiv preprint doi: https://doi.org/10.1101/2021.10.13.21264968; this version posted October 14, 2021. The copyright holder for this preprint (which was not certified by peer review) is the author/funder, who has granted medRxiv a license to display the preprint in perpetuity. This article is a US Government work. It is not subject to copyright under 17 USC 105 and is also made available for use under a CC0 license.

Supplementary Figure 3a.

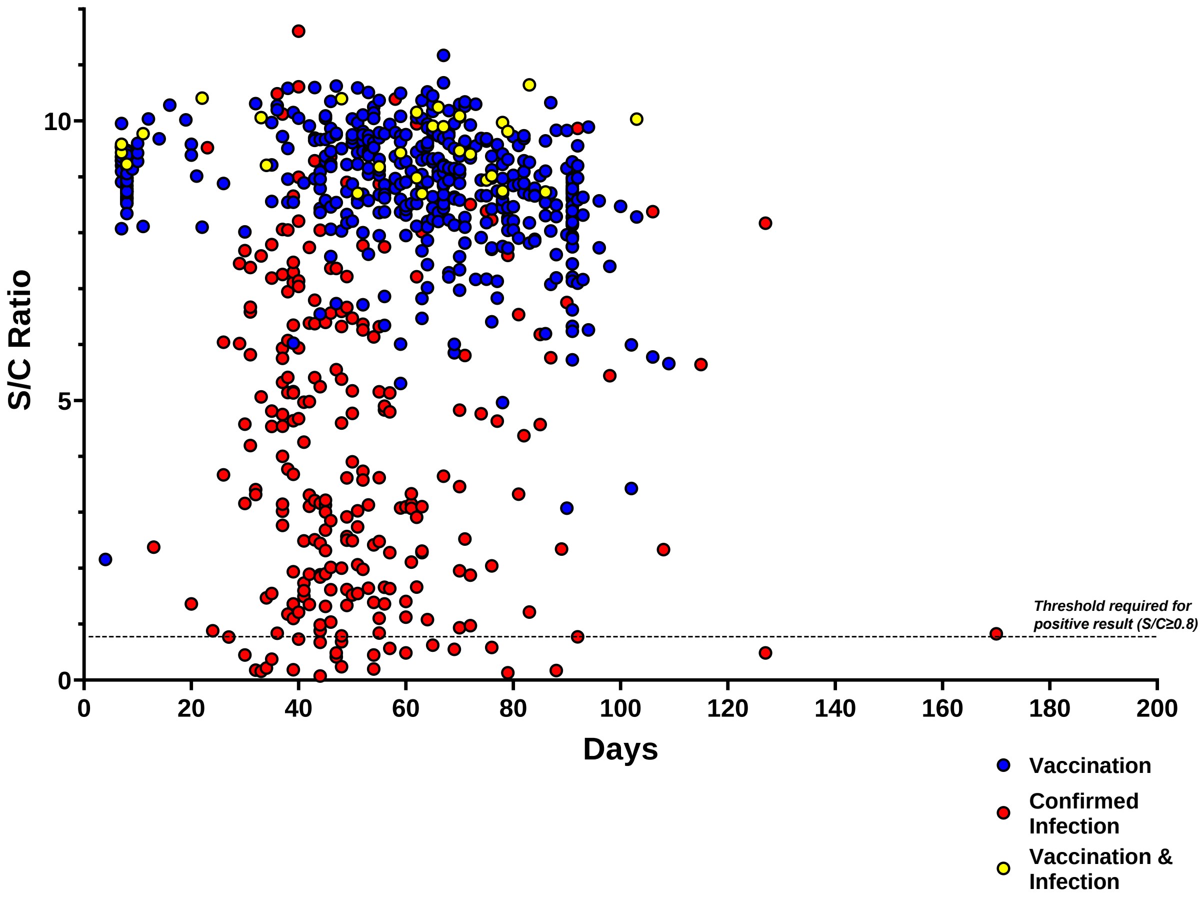

medRxiv preprint doi: https://doi.org/10.1101/2021.10.13.21264968; this version posted October 14, 2021. The copyright holder for this preprint (which was not certified by peer review) is the author/funder, who has granted medRxiv a license to display the preprint in perpetuity. This article is a US Government work. It is not subject to copyright under 17 USC 105 and is also made available for use under a CC0 license.

Supplementary Figure 3b.

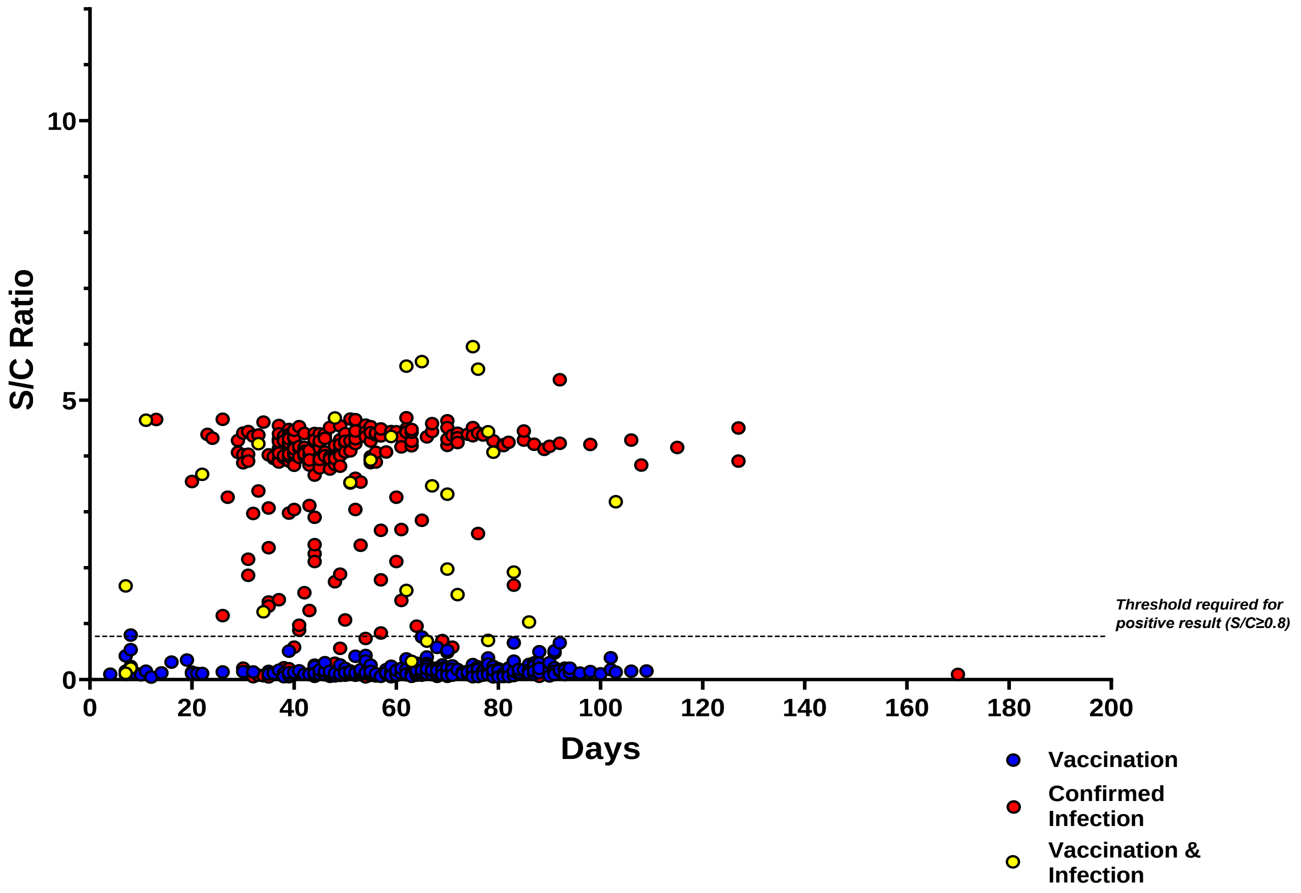

Supplement: 1 [file NIHPP2021.10.13.21264968v1-supplement-1.pdf]
